# A monocentric, open-label randomized standard-of-care controlled study of XONRID®, a medical device for the prevention and treatment of radiation-induced dermatitis in breast and head and neck cancer patients

**DOI:** 10.1186/s13014-020-01633-0

**Published:** 2020-08-13

**Authors:** Rossana Ingargiola, Maria Carmen De Santis, Nicola Alessandro Iacovelli, Nadia Facchinetti, Anna Cavallo, Eliana Ivaldi, Michela Dispinzieri, Marzia Franceschini, Carlotta Giandini, Domenico Attilio Romanello, Simona Di Biaso, Michela Sabetti, Laura Locati, Salvatore Alfieri, Paolo Bossi, Mauro Guglielmo, Fabio Macchi, Laura Lozza, Riccardo Valdagni, Carlo Fallai, Emanuele Pignoli, Ester Orlandi

**Affiliations:** 1 Radiation Oncology Unit 2, Fondazione IRCCS Istituto Nazionale dei Tumori di Milano, Via Venezian 1, 20133 Milan, Italy; 2 Radiation Oncology Unit 1, Fondazione IRCCS Istituto Nazionale dei Tumori di Milano, Milan, Italy; 3 Medical Physics Unit, Fondazione IRCCS Istituto Nazionale dei Tumori di Milano, Milan, Italy; 4 Radiotherapy Department, Sassari Hospital, University of Sassari, Sassari, Italy; 5 Department of Oncology and Hemato-Oncology, University of Milan, Milan, Italy; 6 School of Medicine, University of Milan-Bicocca, Milan, Italy; 7 Post Graduation School in Medical Physics, University of Milan, Milan, Italy; 8 Head and Neck Medical Oncology Unit, Fondazione IRCCS Istituto Nazionale dei Tumori di Milano, Milan, Italy; 9 Medical Oncology, University of Brescia, ASST-Spedali Civili, Brescia, Italy; 10 Oncology-Supportive Care Unit, Fondazione IRCCS, Istituto Nazionale dei Tumori di Milano, Milan, Italy; 11 Helsinn Healthcare SA, Lugano, Switzerland; 12 Prostate Cancer Program, Fondazione IRCCS Istituto Nazionale dei Tumori di Milano, Milan, Italy

**Keywords:** Head and neck cancer, Breast cancer, Acute radiation dermatitis, Skin toxicity, Xonrid®, Skindex-16, Quality of life, Patient-reported outcome measures

## Abstract

**Background:**

This study was an open-label, 2-arms, monocentric, randomized clinical trial comparing Xonrid®, a topical medical device, versus standard of care (SOC) in preventing and treating acute radiation dermatitis (ARD) in Head and Neck Cancer (HNC) and Breast Cancer (BC) patients undergoing radiotherapy (RT).

**Methods:**

Eligible HNC and BC patients were randomized 1:1 to receive Xonrid® + SOC or SOC during RT. Patients were instructed to apply Xonrid® on the irradiated area three times daily, starting on the first day of RT and until 2 weeks after RT completion or until the development of grade ≥ 3 skin toxicity. The primary endpoint was to evaluate the proportion of patients who developed an ARD grade < 2 at the 5th week in both groups. Secondary endpoints were median time to grade 2 (G2) skin toxicity onset; changes in skin erythema and pigmentation and trans-epidermal water loss (TEWL); patient-reported skin symptoms. All patients were evaluated at baseline, weekly during RT and 2 weeks after treatment completion. The evaluation included: clinical toxicity assessment; reflectance spectrometry (RS) and TEWL examination; measurement of patients’ quality of life (QoL) through Skindex-16 questionnaire.

**Results:**

Eighty patients (40 for each cancer site) were enrolled between June 2017 and July 2018. Groups were well balanced for population characteristics. All BC patients underwent 3-Dimensional Conformal RT (3D-CRT) whereas HNC patients underwent Volumetric-Modulated Arc Therapy (VMAT). At week 5 the proportion of BC patients who did not exhibit G2 ARD was higher in Xonrid® + SOC group (*p* = 0.091). In the same group the onset time of G2 ARD was significantly longer than in SOC-alone group (*p* < 0.0491). For HNC groups there was a similar trend, but it did not reach statistical significance. For both cancer sites, patients’ QoL, measured by the Skindex-16 score, was always lower in the Xonrid® + SOC group.

**Conclusion:**

Despite the failure to achieve the primary endpoint, this study suggests that Xonrid® may represent a valid medical device in the prevention and treatment of ARD at least in BC patients, delaying time to develop skin toxicity and reducing the proportion of patients who experienced G2 ARD during RT treatment and 2 weeks later.

**Trial registration:**

The study was approved by the Ethical Committee of Fondazione IRCCS Istituto Nazionale dei Tumori di Milano (INT 52/14 - NCT02261181). Registered on ClinicalTrial.gov on 21st August 2017.

## Introduction

Acute radiation dermatitis (ARD) is a very common side effect of radiotherapy (RT) for breast and head and neck cancer (BC and HNC) patients occurring in about 90–95% of cases [1, 2]. Despite the development of modern techniques, RT induces, through the activation of the inflammatory cascade, skin damage characterized by edema, erythema, dyspigmentation and, in the worst cases, necrosis. Skin reactions occur mostly between the first and the fourth week of treatment, and persist during treatment up to 1-4 weeks after RT end [3, 4]. Its severity depends on RT parameters (dose per fraction, total dose, use of bolus or other beam-modifying devices, radiation type and energy, treatment field size and site treated), the association with chemotherapy (CHT) and patient-related factors, such as comorbidities and individual susceptibility [5].

Severe skin reactions, graded G3 or worse according to Common Toxicity Criteria for Adverse Events (CTCAE) [6] scale, have a profound impact on patients’ quality of life (QoL) and can determine interruptions in RT course, with reduced therapeutic patients’ compliance [7]. Therefore, ARD management represents a priority in setting and delivering supportive care during RT for these patients. Despite a significant development in the prevention and treatment of acute skin reactions, there are no evidences of a clear and standardized management of this side effect [4].

A large number of systemic medications or topical agents have been tested in order to prevent or cure ARD and few of them have been claimed to have favorable effects in managing this toxicity [8–16]. However, recent systematic reviews on ARD in BC and HNC patients suggest that there is still no strong evidence supporting superiority of any specific intervention over another or over the skin care guidelines developed by the Multinational Association of Supportive Care in Cancer (MASCC) [7, 17]. Based on the aforementioned findings, these guidelines could be considered the Standard of Care (SOC) for addressing ARD.

This unmet clinical need leads to develop new products, adopting trial designs which consider physician-reported toxicity and patients’ subjective evaluations to better depict ARD burden [18].

Xonrid® is an EC-marked medical device, class IIa, specifically designed for ARD. It is a topical water-based gel for the prevention and treatment of skin symptoms such as erythema, itching, burning sensation and pruritus, induced by RT. When applied to the target skin areas, Xonrid® forms a protective film, thereby reducing trans-epidermal water loss (TEWL) and increasing moisturizing. Moreover, it promotes the healing process by restoring the physiological hydration levels of the affected skin areas.

In our previous pilot study on 42 HNC patients receiving curative conventional fractionated RT and chemotherapy (CHT), Xonrid® use resulted in a decreased incidence of G3 toxicity, according to CTCAE criteria, and a delay in the development of G2 toxicity when compared to the data deriving from an historical cohort [19]. Indeed, at the 5th week of RT, patients with G2 and G3 dermatitis were about 52 and 10% in the historical cohort and 15 and 2% in the pilot study, respectively. Finally, Xonrid® resulted well tolerated, safe, and effective in minimizing and delaying severe ARD [20].

Thus, we designed a randomized trial aimed to evaluate the effectiveness of Xonrid® compared to Standard of Care (SOC) [17] in preventing and treating ARD up to G2 (according to CTCAE) in BC and HNC patients.

## Materials and methods

### Study design

This study was approved by the local Ethical Committee (INT 52/14 - NCT02261181) and conducted according to the ethical principles of the declaration of Helsinki. All patients provided a written informed consent, which they could withdraw at any time without prejudicing further medical care.

The design was a monocentric, open label, randomized, standard-of-care controlled, post-marketing clinical investigation. Eligible BC and HNC patients were randomized in a ratio of 1:1 to receive: Xonrid® gel + SOC preemptive treatment according to MASCC guidelines (Group A) versus SOC preemptive treatment according to MASCC guidelines alone [17] (Group B). This trial primary objective was to evaluate the proportion of patients without G2 ARD (ARD <G2) at the 5th RT week.

### Patient selection

Patients with non metastatic BC and HNC (from oropharynx, nasopharynx, larynx, hypopharynx, paranasal sinuses and salivary glands sites) treated with postoperative or definitive RT were eligible for this study.

As inclusion criteria, all patients had to be older than 18 years old and BC patients also younger than 60 years old; performance status had to be < 2; HNC patients with concurrent CHT were accepted; patients had to be willing and able to give signed informed consent and, in the opinion of the investigators, able to comply with the planned test procedure. Criteria leading to exclusion were: pregnancy and lactation; concurrent anti-epidermal growth factor receptor (EGFR) therapy (i.e. Cetuximab); previous RT on the head and neck or breast and thorax areas; superficial disease site requiring use of a tissue-equivalent bolus; cutaneous or connective diseases; systemic diseases delaying the skin healing process (e.g. diabetes mellitus, severe renal failure), presence of rashes or unhealed wounds in the radiation field, recent sun exposure or mental conditions that could adversely affect patients’ adherence.

### Statistical analysis

The clinical investigation hypothesis was that Xonrid®, applied together with SOC preemptive treatment (according to MASCC guidelines) would slow down the progression of radiation dermatitis to G2, thus reducing the number of G2 events at the 5th week.

In a previous observational study focusing only on HNC, 38.2% of the patients treated with SOC did not reach G2 ARD at the 5th week [17]. In our pilot study, always focused on HNC, the proportion of patients treated with Xonrid® and SOC preemptive treatment not reaching G2 ARD at the 5th week was 82.9% [20]. We started from the assumption that similar proportions would be observed in the present study, leading to an estimate of 36 patients (18 per treatment group) needed to achieve a power of 80%, with α = 0.05. Four more patients were enrolled, considering a 10% dropout rate. In total, 80 patients were enrolled (40 patients with HNC and 40 with BC).

To compare demographic and baseline characteristics between treatment groups, chi-square or t-tests were used for discrete and continuous variable, respectively.

The statistical analyses were performed using SAS 9.4 for Windows (SAS Institute Inc., Cary, NC, USA).

### Endpoints of the study

#### Primary endpoint

The primary objective of this clinical investigation was *to evaluate the proportion of patients without G2 ARD (ARD grade < 2) at the 5th week.* The proportion of subjects with this feature in the two study arms was compared using chi-square test. Logistic regression was applied to obtain the Odds Ratio (OR), with 95% Confidence Intervals (95% CI).

#### Secondary endpoints

The median time to G2 ARD development was analyzed using Kaplan-Meier method. Comparisons between the treatment groups were performed using log-rank tests.

The proportion of patients without G2 ARD was assessed at the 6th week and 2 weeks after the last irradiation for both cancer sites and also at the 7th week for HNC; chi-square test was performed to compare this proportion between the treatment groups.

Logistic regression was applied to obtain ORs, with 95% CIs, and to eventually adjust for covariates.

The worst skin toxicity during RT treatment and until 2 weeks after the last irradiation was compared between the treatment groups using ANOVA.

The mean and worst scores of Skindex-16 questionnaire were compared between the treatment groups using ANCOVA, to adjust the estimates for baseline values.

The changes in skin erythema and pigmentation were graphically described reporting the ITA (Individual Typology Angle) degrees throughout the study, while TEWL changes were described plotting the Evaporation rate values (ERV, measured in g/m^2^h) throughout the study. ANCOVA test was used to compare those changes between the treatment groups.

The patients’ global satisfaction with the treatment, assessed by Likert scale, was compared between the treatment groups using Kruskal-Wallis test.

### Treatments

#### Radiotherapy

All patients received computed tomography simulation in treatment position. HNC patients received Volumetric-Modulated Arc Therapy (VMAT) with two or three coplanar arcs. In definitive setting, RT total doses were 70, 60 and 50–54 Gy to high, intermediate and low risk volume, respectively; in postoperative cases total dose to the corresponding volumes were 66, 56–60 and 54 Gy, respectively. In all cases conventional or moderately accelerated fractionation (1.80–2.15 Gy/die) was used, with an overall treatment time of 6. 5-7 weeks. According to histology, stage and pathology reports, patients could receive concomitant platinum-based CHT [21, 22].

Concerning BC patients selection, only patients younger than 60 years old were included because during the study period, according to institutional policy and international guidelines, patients aged 60 years or more received hypofractionated whole breast radiotherapy [23] In this trial all patients received conventional fractionated RT (2 Gy per fraction) with three dimensional conformal technique (3D-CRT): two isocentric tangential fields with wedge filter and/or multileaf collimator were used to optimize dose distribution to the whole breast volume. In general, two photon beams were also used sequentially for the boost volume. Prescription dose was 50 Gy in 25 consecutive daily fractions, followed by a boost dose of 10 Gy in 5 fractions, with a 6 weeks overall treatment time.

In order to analyze the relationship between RT dose distribution and patients’ superficial skin layer, a structure called skin volume (Vskin) was semi-automatically created and consisted of a 3 mm layer below the external patient surface (Body) for both HNC and BC patients. The dosimetric parameter collected was Vskin_X_, representing the volume receiving a dose “X “in the range 5-50 Gy and 5-70 Gy (5Gy steps) for BC and HNC, respectively. In order to minimize the potential bias introduced by differences in prescription dose and number of fractions among BC and HNC patients (25 versus 33, respectively), the X dose for HNC was normalized to 33 fractions and multiplied by 25 (i.e. x/fraction_number*25). As for BC, the boost was not considered since the endpoint of the study was G2 evaluation at the 5th week.

#### Skin care

Enrolled patients were randomized in a ratio of 1:1 to receive Xonrid® gel and SOC preemptive treatment according to MASCC guidelines (group A) or SOC-only (group B) [17].

Xonrid® was given in topical gel formulation. Patients were instructed to apply it on the irradiated area three times daily: the first application 1-2 h after the morning RT session, the second in the early afternoon and the third in the evening, also during the weekends (when subjects did not receive RT), starting from the first day of RT. The estimated amount of product to be used at each application was 12-18 dispensed doses for patients affected by HNC and 6 puffs for those affected by BC.

Both treatments continued until 2 weeks after RT completion or until the development of G ≥ 3 skin toxicity. The use of other topical medications, in particular topical steroid creams, was not allowed.

When a patient (both HNC and BC) developed G2 ARD, she/he continued the treatment (Xonrid® + SOC) until G3 toxicity occurred (Group A). When a patient developed G3 ARD (both Groups A and B), she/he interrupted the study treatment and participation and further care for that subject was chosen by the Investigators.

#### Tools for ARD assessment

All patients were evaluated at baseline, at weekly intervals during RT and 2 weeks after treatment completion. The evaluation consisted of a physician-assessed toxicity using CTCAE scale v.4.0, patient-reported outcomes (PRO) measured using the Skindex-16 questionnaire**,** reflectance spectrometry (RS) examination to objectively in vivo measure skin erythema and pigmentation and TEWL examination. Specifically, for each patient skin reflectance measurements were acquired in vivo by a spectrophotometric imaging system (SkinColorCatch, by Delfin Technologies Ltd. – Finland) in three different predefined regions within RT-treated area – always on flat skin regions and in absence of hair or nevus. A control measurement was also done in a specific contralateral area. Higher values were related to higher pigmentation levels and erythema.

The TEWL exam was done at the same time-points and skin areas as the spectrophotometric exam, using the VapoMeter instrument (by Delfin Technologies Ltd. – Finland). Higher values were related to higher TEWL.

#### Compliance

Compliance to the treatment was verified when the patients brought back the used/unused products by weighting the bottles. A cross check was done with patient’s diary information.

#### Patient global satisfaction

Patient global satisfaction was assessed through a 5-point Likert scale (very poor, poor, medium, good, very good) at the end of the RT sessions and two weeks after the last irradiation.

## Results

### Study population

Eighty patients, 40 for each cancer site (HNC and BC), were enrolled since June 16th 2017 to July 25th 2018, randomized to receive SOC or Xonrid® + SOC. Characteristics and treatment details of the study population are shown in Tables 1 and 2. These parameters resulted no significantly different in the two treatment arms for HNC and BC patients groups. Twenty-two out of 40 HNC patients completed the study, 8 (40%) and 14 (70%) for the SOC and Xonrid® + SOC groups, respectively. Whereas, 29 out of 40 BC patients completed the study, 12 (60%) and 17 (85%) in the SOC and Xonrid® + SOC treatment groups, respectively. Main dropout reason was the development of G3 skin toxicity for both cancer sites, as it is shown in Table 3. After RT completion, all patients continued with the assigned treatment for further 2 weeks.

**Table 1:** Demographics and Treatment details of Breast Cancer Study Population

Characteristics		SOC (***N*** = 20)	Xonrid® + SOC (N = 20)	***p***-Value
***Gender***	Females	20 (100.0%)	20 (100.0%)	n.a.
***Age***	N	20	20	0.7
	Mean (SD)	49.01 (4.54)	49.56 (4.80)	
	Median	48.99	49.03	
	Range	38.26–57.75	40.38–57.94	
***ECOG performance status***	0	20 (100.0%)	20 (100.0%)	n.a.
***Concomitant medications for comorbidities***	Yes	12 (60.0%)	9 (45.0%)	0.3
	No	8 (40.0%)	11 (55.0%)	
***Histology***	DCIS	2 (10.0%)	3 (15.0%)	0.4
	Invasive Ductal Carcinoma	18 (90.0%)	17 (85%)	
***Stage TNM VIII Edition***	DCIS	2 (10.0%)	3 (15.0%)	0.5
	IA	11 (55.0%)	16 (80.0%)	
	IB	1 (5%)	1 (5%)	
	II A	3 (15%)		
	IIB	3 (15%)		
***Prescribed total dose/ N° of fractions***	50 / 25	2 (10.0%)	3 (15.0%)	0.6
	60 / 30	18 (90.0%)	17 (85.0%)	
***Baseline Erythema assessment - Skin toxicity grade***	No erythema	20 (100.0%)	20 (100.0%)	0.3
***SKINDEX-16 - Symptom score***	N	20	20	0.3
	Mean (SD)	0.06 (0.23)	0.16 (0.40)	
	Median	0.00	0.00	
	Min - Max	0.00–1.00	0.00–1.50	
***SKINDEX-16 - Emotional score***	N	20	20	0.9
	Mean (SD)	0.19 (0.46)	0.16 (0.31)	
	Median	0.00	0.00	
	Min - Max	0.00–1.71	0.00–1.00	
***SKINDEX-16 - Functional score***	N	20	20	0.6
	Mean (SD)	0.08 (0.28)	0.16 (0.54)	
	Median	0.00	0.00	
	Min - Max	0.00–1.20	0.00–2.40

**Table 2:** Demographics and Treatment details of Head and Neck Cancer Study Population

Characteristics		SOC (N = 20)	Xonrid® + SOC (N = 20)	P-Value
***Gender***	Females	4 (20.0%)	4 (20.0%)	1.0
	Males	16 (80.0%)	16 (80.0%)	
***Age***	N	20	20	0.6
	Mean (SD)	57.49 (12.34)	55.73 (12.46)	
	Median	58.36	59.18	
	Range	32.32–79.24	24.70–76.61	
***ECOG performance status***	0	20 (100.0%)	20 (100.0%)	n.a.
***Concomitant medications for comorbidities***	Yes	10 (50.0%)	9 (45.0%)	0.7
	No	10 (50.0%)	11 (55.0%)	
***Cancer Subsite***	Hypopharynx		1 (5.0%)	0.07
	Larynx	2 (10.0%)	2 (10.0%)	
	Nasopharynx	10 (50.0%)	5 (25.0%)	
	Oral Cavity	4 (20.0%)		
	Oropharynx	4 (20.0%)	10 (50.0%)	
	Paranasal Sinuses		2 (10.0%)	
***Stage (TNM VIII Edition)***				
*Clinical*		14 (70%)	18 (90%)	0.5
	I/II	1 (7.2%)	2 (11.1%)	
	III/IV	13 (92.8%)	16 (88.9%)	
*Pathological*		6 (30%)	2 (10%)	
	I/II	0	0	
	III/IV	6 (100%)	2 (100%)	
***Aim of radiation treatment***				
*Definitive*		14 (70.0%)	16 (80.0%)	0.5
*Postoperative*		6 (30.0%)	4 (20.0%)	
***Prescribed total dose/ N° of fractions***				
*HD-PTV Total planned dose / N° of fractions*	60.00 / 30.00	2 (10.0%)	1 (5.0%)	0.6
	64.50 / 30.00	1 (5.0%)		
	66.00 / 33.00	3 (15.0%)	2 (10.0%)	
	69.96 / 33.00	14 (70.0%)	16 (80.0%)	
	70.00 / 35.00		1 (5.0%)	
*ID-PTV Total planned dose / N° of fractions*	0.00	7 (36.8%)	8 (42.1%)	0.5
	59.40 / 33.00	10 (52.6%)	1 (52.6%)	
	60.00 / 30.00	1 (5.3%)		
	66.00 / 33.00	1 (5.3%)		
*LD-PTV Total planned dose / N° of fractions*	54.00 / 30.00	3 (15.0%)	1 (5.0%)	0.4
	56.00 / 35.00		1 (5.0%)	
	56.10 / 33.00	14 (70.0%)	17 (85.0%)	
	59.40 / 33.00	3 (15.0%)	1 (5.0%)	
***Baseline Erythema assessment - Skin toxicity grade***	No erythema	20 (100.0%)	20 (100.0%)	n.a.
***SKINDEX-16 - Symptom score***	N	19	20	0.3
	Mean (SD)	0.00 (0.00)	0.05 (0.22)	
	Median	0.00	0.00	
	Min - Max	0.00–0.00	0.00–1.00	
***SKINDEX-16 - Emotional score***	N	19	20	0.02*
	Mean (SD)	0.07 (0.20)	0.79 (1.27)	
	Median	0.00	0.00	
	Min - Max	0.00–0.71	0.00–5.14	
***SKINDEX-16 - Functional score***	N	19	20	0.2
	Mean (SD)	0.08 (0.37)	0.56 (1.39)	
	Median	0.00	0.00	
	Min - Max	0.00–1.60	0.00–6.00	
Concomitant Chemotherapy	Yes	16 (80.0%)	17 (85.0%)	0.7
	No	4 (20.0%)	3 (15.0%)

**Table 3:** Dropout reason in BC and HNC patients

Reason for withdrawal	BC		HNC	
	***SOC (N = 8)***	***Xonrid® + SOC (N = 3)***	***SOC (N = 12)***	***Xonrid® + SOC (N = 6)***
***Grade > = 3 skin toxicity***	4 (50.0%)	1 (33.3%)	5 (41.7%)	6 (100.0%)
***Adverse event***	1 (12.5%)	1 (33.3%)	1 (8.3%)	
***Lost to follow-up***	1 (12.5%)	1 (33.3%)	3 (25.0%)	
***Consent withdrawal***	1 (12.5%)		3 (25.0%)	
***Lack of compliance to study treatment or assessment***	1 (12.5%)			

The compliance with the study treatment (Xonrid® + SOC) was 96.5 and 93% during RT and 97 and 100% 2 weeks after RT completion for BC and HNC patients, respectively.

### Primary endpoint

The proportion of BC patients that did not reach G2 skin toxicity at the 5th week was much higher in the Xonrid® + SOC group than in the SOC-only group (55.6% vs. 27.8%) and the difference was close to statistical significance (*p* = 0.091). With regard to HNC population, the proportion of patients not reaching G2 ARD at the 5th week was slightly higher in the SOC-alone group than in the one treated with Xonrid® + SOC (68.8% vs. 65%), but the difference was not statistically significant (*p* = 0.8) (Table 4).

**Table 4:** Number and proportion of patients that reached and not reached G2 at week 5

Visit	Statistics	BC		HNC	
		***SOC***	***Xonrid® + SOC***	***SOC***	***Xonrid® + SOC***
**WEEK 5**	NO G2	5 (27.8%)	10 (55.6%)	11 (68.8%)	13 (65.0%)
	G2+	13 (72.2%)	8 (44.4%)	5 (31.3%)	7 (35.0%)
	p-value (Chi-square)		0.09		0.8
**Logistic regression**	Odds ratio		0.31		1.18
	95% CI		0.08–1.23		0.29–4.81
	p-value		0.1		0.8

### Secondary endpoints

**Fig. 1:**
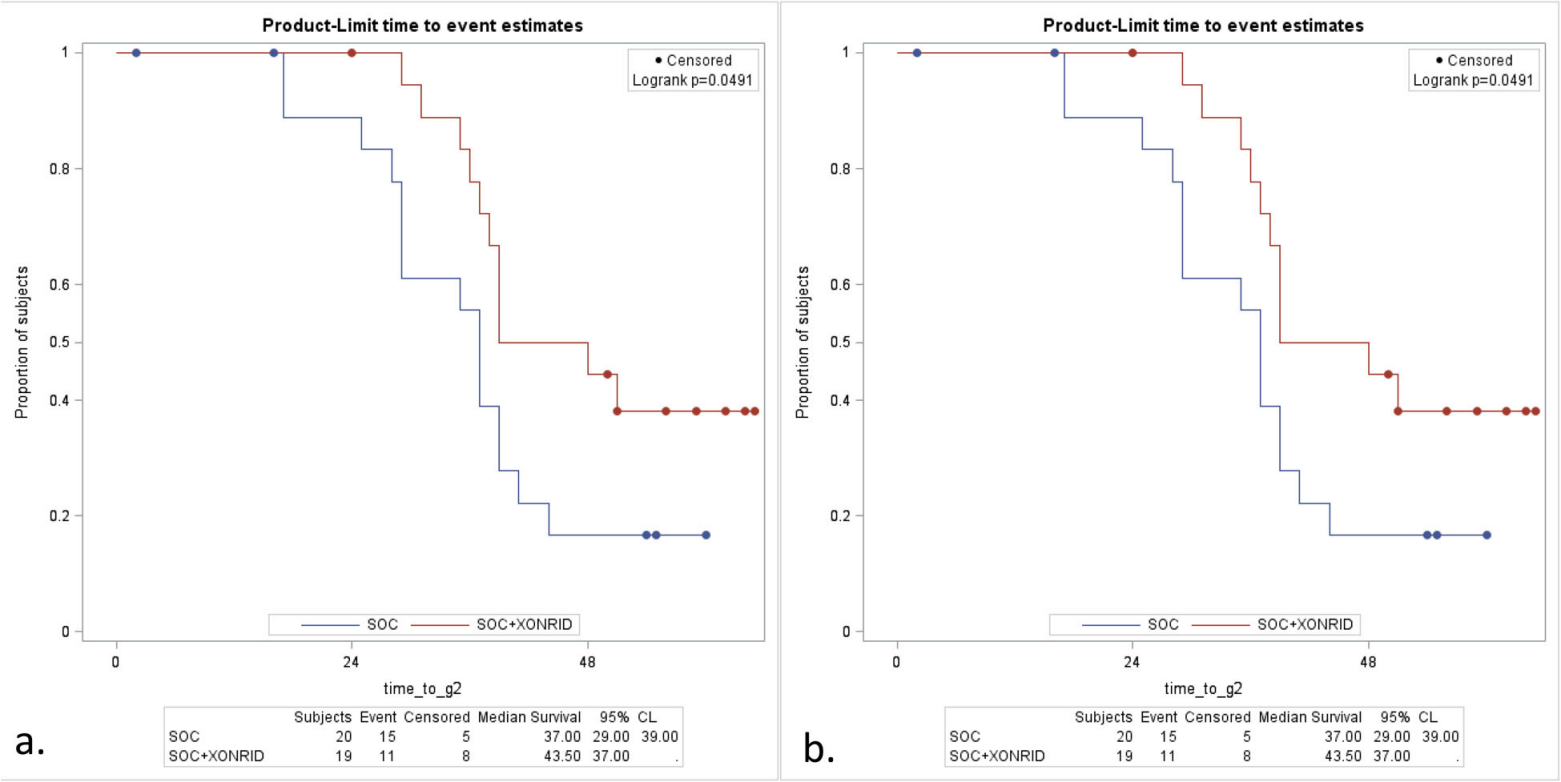
Median time to G2 ARD in **a**) BC patients and **b**) HNC patients

Median time to G2 ARD was significantly higher among BC patients treated with Xonrid® + SOC compared to SOC-only (*p* < 0.049) (Fig. 1a). A similar favourable trend has been observed in HNC patients, but it did not reach statistical significance (Fig. 1b).

**Fig. 2:**
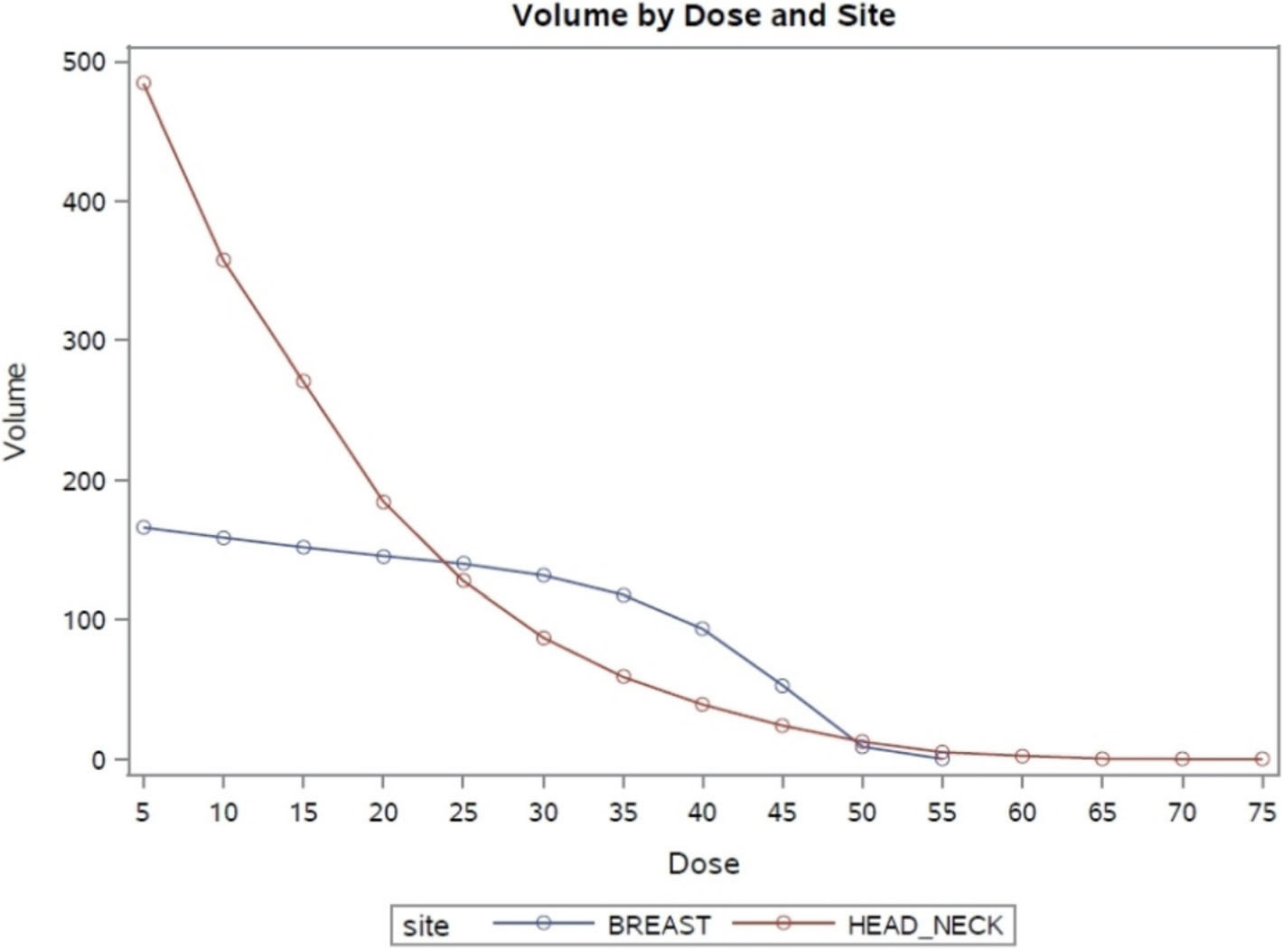
Skin volume (Vskin) involved in the higher dose levels for BC and HNC patients

We also observed that G2 ARD appeared later in HNC compared to BC, mainly around the 6th week. This result could be justified by the difference in the skin volume involved in the higher dose levels, as it is shown in Fig. 2.

For both cancer sites, no difference was detected between the treatment groups in the proportion of patients reaching G2 at the 6th week (and 7th for HNC patients) and 2 weeks after the end of RT (Table 5).

**Table 5:** G2 at week 6, 7 and 2 weeks after RT in BC and HNC patients

Visit	Statistics	BC		HNC	
		***SOC***	***Xonrid® + SOC***	***SOC***	***Xonrid® + SOC***
WEEK 6	NO G2	3 (16.7%)	7 (38.9%)	6 (37.5%)	9 (45.0%)
	G2+	15 (83.3%)	11 (61.1%)	10 (62.5%)	11 (55.0%)
	p-value (Chi-square)		0.1		0.6
Logistic regression	Odds ratio		0.31		0.73
	95% CI		0.07–1.50		0.19–2.81
	p-value		0.1		0.6
WEEK 7	NO G2			3 (21.4%)	5 (25.0%)
	G2+			11 (78.6%)	15 (75.0%)
	p-value (Chi-square)				0.8
Logistic regression	Odds ratio				0.82
	95% CI				0.16–4.17
	p-value				0.8
2 WEEKS AFTER END OF TREATMENT	NO G2	3 (16.7%)	6 (35.3%)	12 (100.0%)	15 (100.0%)
	G2+	15 (83.3%)	11 (64.7%)		
	p-value (Chi-square)		0.2		
Logistic regression	Odds ratio		0.37		
	95% CI		0.07–1.80		
	p-value		0.2		

For the whole population, regardless of cancer site, Skindex-16 scores at each visit were always lower in the group treated with Xonrid® + SOC (n.s.). Same results were observed for subscales (Additional files 1 and 2).

**Fig. 3:**
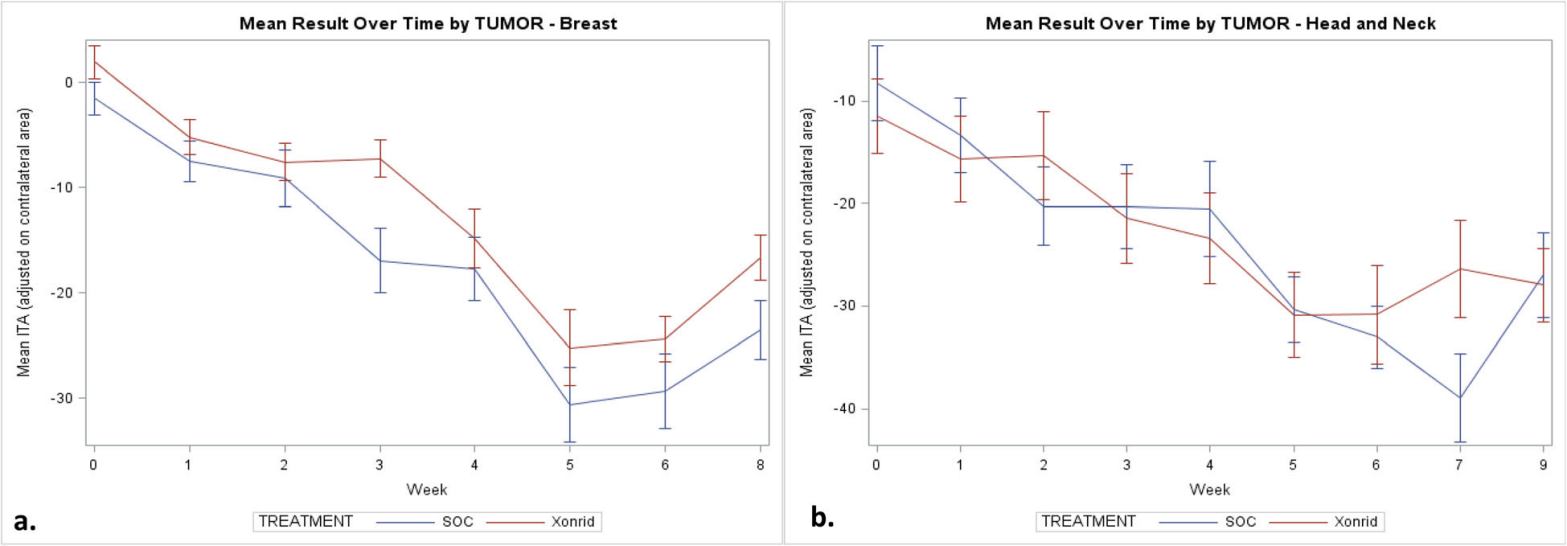
Mean ITA observed during the study for **a**) BC parients and **b**) HNC patients

For BC patients, mean ITA at each timepoint, adjusted for contralateral area, was always higher in the Xonrid® + SOC treatment group. The difference was statistically significant at the 3rd week (*p* < 0.01) and borderline at 2 weeks after RT completion (*p* = 0.052) (Fig. 3a).

For HNC patients, mean ITA at each timepoint, adjusted for contralateral area, was often higher in the Xonrid® + SOC treatment group, but the differences were not statistically significant. The highest difference was observed at the 7th week, but it was not statistically significant due to the low number of patients examined during this visit (8 and 13 patients in the SOC and Xonrid® + SOC treatment groups, respectively) (Fig. 3b).

Mean TEWL at each timepoint, adjusted for contralateral area, was often lower in the Xonrid® + SOC treatment group. The difference was statistically significant at the 3rd and 6th week (*p* < 0.05), if not adjusted for baseline values, and 2 weeks after RT end when adjusted for baseline values (p < 0.05).

### Safety analysis

All adverse events (AEs) observed were classified as mild to moderate. Only one of them (pruritus) was considered possibly related to the treatment with Xonrid® + SOC (Additional files 3 and 4).

## Discussion

Despite the improvement in radiation techniques, ARD still represents a very common acute side effect during and immediately after the end of RT.

Nowadays, despite the numerous trials in literature analyzing a wide variety of topical pharmaceuticals and non-pharmaceutical agents, there is still no standard approach for addressing ARD. In their metanalysis, Haruna et al. showed the efficacy of topical corticosteroids in ARD treatment for BC patients [3]. Use of corticosteroid is currently under evaluation in HNC, however its prolonged use is associated with important side effects such as skin thinning. Ghasemi et al. investigated the topical use of atorvastatin in a recent randomized trial, reporting an improvement in associated symptoms but not in ARD reduction [14]. Many other pharmaceuticals and biological agents were investigated (e.g. sucralfate, trolamine, EGF based-cream) as well as non-pharmeceutical agents, but none of these proved to be effective in ARD prevention or treatment. Starting from this lack of evidence in literature, we decided to test Xonrid®, a new medical device. This work reports the results obtained with Xonrid® and SOC, compared to SOC alone, in a prospective randomized trial performed on BC and HNC patients addressing ARD. For both cancer sites the proportion of patients not reaching G2 ARD at the 5th week (the primary endpoint) was higher (ns) in the group treated with Xonrid® + SOC than SOC alone. This result is not in line with our previous pilot study on HNC, when we were able to report a significant advantage in using Xonrid®. However, we must underline that was a single-arm pilot study and the results were compared with historical control cohorts [19].

As regards the secondary endpoints, median time to G2 ARD was significantly higher among BC patients treated with Xonrid® + SOC compared to SOC alone (*p* < 0.049) (Fig. 1a), likely suggesting that G2 toxicity arose later among patients treated with Xonrid® + SOC with respect to the others. These results are encouraging, but certainly deserve further studies with larger cohorts in order to draw definitive conclusions, also in a time when hypofractionated whole-breast RT is becoming increasingly employed [24].

At the time the study began hypofractionation was not a standard option, at our Institution, for young BC patients, who represent the majority of our BC population [25]. The use of beam-modifying devices, like boluses, known to improve ARD, was an exclusion criteria, mostly for reducing the inhomogeneity in the study population.

As shown in literature the grade and severity of ARD is less important in hypofractionated regimen, probably making the use of topical devices less useful [26].

Conversely a topical product could be useful in the prevention and treatment of this side effect in the proton era, since the incidence of ARD seems to be higher as reported in a recent paper by the University of Maryland, showing a higher rate of ARD for patients treated with protons [27].

In contrast, there was no difference between the two HNC patients’ groups in the median time to G2 ARD. This could be related to the skin volume involved during RT. For HNC patients, skin volumes receiving high dose levels are smaller compared to BC patients, but at the end of the RT treatment some portions of the skin receive higher total doses (55–75 Gy). This could justify the delay of G2 ARD onset but also the reduced effect (or efficacy) of Xonrid® + SOC. In the HNC cohort, in fact, we observed that G2 ARD arose on average later during treatment, with respect to BC patients. It is known that ARD development and gravity depends on a variety of treatment- and patient-related factors. An explanation could be the different RT technique used in BC and HNC patients. In our study, BC patients were treated with 3D-CRT, that still represents a standard in the management of BC without involved nodes, cardiac comorbidities or particular anatomical conformation, while HNC patients received VMAT, a technique that produces better dose homogeneity than conventional RT.

Perhaps Xonrid® is more effective in reducing skin toxicity in patients undergoing whole breast irradiation with standard-field techniques, in which dose homogeneity may be less optimal. The use of breast Intensity-Modulated RT (IMRT) was recently found to reduce the incidence of moist desquamation relative to the use of standard opposed-wedge techniques in a double-blind multicenter phase III trial [28].

Among other causes of skin-reactions severity observed in HNC patients, Lee et al. (2002) mentioned the bolus effect of thermoplastic masks, the use of multiple tangential beams in IMRT and the inclusion of the inner part of the skin into the target volume, because of its proximity to lymph nodal target areas [29].

Moreover, we need to consider that the majority of HNC patients underwent concomitant CHT. Previous findings indicated that concomitant CHT-RT is more likely to induce certain acute and late severe toxicities than RT alone [30].

Other factors influencing skin toxicity are classified like intrinsic or patient-related factors: malnutrition, smoking habit, UV exposure - more typical of HNC than BC patients - as well as genetic and hormonal factors. About the last ones, another reason for the different behavior between breast and head and neck skin could be ascribed to gender-based physiological differences. It has been demonstrated that different levels of sex and stress hormones could play a role in the differences between male and female skin [31]. This could reflect into dissimilar skin response to radiation. In particular, some Authors have found that male skin is more prone to malignancies because of its higher immunosuppression tendency, whereas female skin is more prone to cutaneous disorders, making it more reactive to exogenous insults [32].

In addition, the higher number of dropouts in the HNC cohort may have evidenced a difference in favor of the BC cohort, bound to disappear when investigating and analyzing larger patient groups.

A strength of our study is the assessment of toxicity through several procedures, both subjective and objective: an unprecedented approach aimed at making this study a pilot investigation in this domain. Xonrid® effects were measured, in addition to clinical evaluation, through a PRO measure using the Skindex-16 questionnaire and by detecting changes in skin erythema and pigmentation, according to ITA, and changes in TEWL.

Few studies in literature could be found correlating QoL to ARD, mostly regarding HNC patients and using heterogeneous methods to evaluate QoL. In 2014 Chan et al. evaluated QoL through Skindex-16 questionnaire, showing no difference in the impact of NOVA cream compared to aqueous cream in HNC patients. The phase III trial RTOG 97–13 found that Biafine did not reduce skin toxicity or improve QoL (using Spitzer quality-of-life questionnaires) compared with best SOC during adjuvant RT for BC [33].

In our study, BC patients treated with Xonrid® + SOC appeared to perform better than patients treated with SOC alone in terms of Skindex-16 scores, but the values did not reach statistical significance. Instead, HNC patients treated with Xonrid® + SOC did not show any difference in terms of skin-related QoL with respect to SOC alone group. However, Skindex-16 scores at each visit were quite lower in the Xonrid® + SOC group (n.s.).

Both groups did not show any difference in ITA and TEWL values. We could therefore imply that those methods need refinement for being considered as ARD objective assessments.

## Conclusion

In conclusion, although we were not able to demonstrate a reduction in the proportion of patients not reaching G2 ARD at the 5th week of RT (our primary endpoint), this study suggests that Xonrid® may present considerable advantages in the prevention and treatment of ARD at least in BC patients. The main limitation of this study is the small number of patients available for statistical analysis, since the number of dropout patients was higher than the 10% estimated in the trial design. This could have affected the results, particularly for the HNC cohort. Therefore, confirmation is needed in a study including a larger number of patients in both treatment and control groups.

## Supplementary information

**Additional file 1.** Skindex-16 in BC patients.

**Additional file 2.** Skindex-16 in HNC patients.

**Additional file 3.** AEs in BC patients.

**Additional file 4.** AEs in HNC patients.

## Data Availability

The datasets used and/or analyzed are available from Helsinn Healthcare SA on reasonable request.

## Abbreviations

ARD: Acute radiation dermatitis
AEs: Adverse events
BC: Breast cancer
CT: Chemotherapy
CI: Confidence intervals
CT: Computerized tomography
CTCAE: Common terminology criteria for adverse events
EGFR: Epidermal growth factor receptor
ERV: Evaporation rate values
HNC: Head and neck cancer
IMRT: Intensity-modulated radiotherapy
ITA: Individual typology angle
MASCC: Multinational association of supportive care in cancer
OR: Odds ratio
PRO: Patient-reported outcomes
QoL: Quality of life
RT: Radiotherapy
RS: Reflectance spectrometry
SOC: Standard of care
TEWL: Trans-epidermal water loss
3D-CRT: Three dimensional conformal radiotherapy
VMAT: Volumetric-modulated arc therapy
Vskin: Skin volume
