# Supplementary material for: A monocentric, open-label randomized standard-of-care controlled study of XONRID®, a medical device for the prevention and treatment of radiation-induced dermatitis in breast and head and neck cancer patients
Source: Radiat Oncol. 2020 Aug 13;15:193. doi: 10.1186/s13014-020-01633-0 (PMC7427075; doi:10.1186/s13014-020-01633-0)
Supplement: Supplementary file 1 — Additional file 1. Skindex-16 in BC patients. [file 13014_2020_1633_MOESM1_ESM.docx]

**Additional file 1**

**Skindex-16 in BC patients**

| **Endpoint** | **Statistic** | **SOC (N=20)** | **Xonrid®+SOC (N=20)** |
| --- | --- | --- | --- |
| *Week 5* | N | 13 | 17 |
|  | Mean (SD) | 1.63 (2.20) | 1.22 (1.27) |
|  | Median | 0.50 | 1.00 |
|  | Min - Max | 0.00 / 6.00 | 0.00 / 4.00 |
| *T-test* | Adjusted mean (SE) | 1.63 (0.48) | 1.22 (0.42) |
|  | Treatment difference |  | -0.41 |
|  | 95% CI |  | -1.72 / 0.89 |
|  | p-value |  | 0.521 |
|  |  |  |  |
| *ANCOVA model* | Adjusted mean (SE) | 1.68 (0.49) | 1.18 (0.42) |
|  | Treatment difference |  | -0.50 |
|  | 95% CI |  | -1.83 / 0.84 |
|  | p-value |  | 0.451 |
|  |  |  |  |
| *Week 6* | N | 13 | 17 |
|  | Mean (SD) | 1.60 (1.69) | 1.54 (1.54) |
|  | Median | 1.25 | 1.00 |
|  | Min - Max | 0.00 / 5.50 | 0.00 / 4.75 |
| *T-test* | Adjusted mean (SE) | 1.60 (0.45) | 1.54 (0.39) |
|  | Treatment difference |  | -0.05 |
|  | 95% CI |  | -1.26 / 1.16 |
|  | p-value |  | 0.931 |
|  |  |  |  |
| *ANCOVA model* | Adjusted mean (SE) | 1.73 (0.45) | 1.44 (0.39) |
|  | Treatment difference |  | -0.28 |
|  | 95% CI |  | -1.55 / 0.98 |
|  | p-value |  | 0.649 |
|  |  |  |  |
| *2 Weeks after RT end* | N | 17 | 20 |
|  | Mean (SD) | 1.32 (1.91) | 0.78 (0.83) |
|  | Median | 0.50 | 0.63 |
|  | Min - Max | 0.00 / 5.25 | 0.00 / 3.25 |
| *T-test* | Adjusted mean (SE) | 1.32 (0.35) | 0.78 (0.32) |
|  | Treatment difference |  | -0.55 |
|  | 95% CI |  | -1.51 / 0.41 |
|  | p-value |  | 0.252 |
|  |  |  |  |
| *ANCOVA model* | Adjusted mean (SE) | 1.34 (0.35) | 0.76 (0.33) |
|  | Treatment difference |  | -0.59 |
|  | 95% CI |  | -1.57 / 0.40 |
|  | p-value |  | 0.236 |
| *Worst Skindex-16 score* | N | 20 | 20 |
|  | Mean (SD) | 2.16 (2.18) | 1.80 (1.45) |
|  | Median | 0.88 | 1.13 |
|  | Min - Max | 0.00 / 6.00 | 0.00 / 4.75 |
| *T-test* | Adjusted mean (SE) | 2.16 (0.41) | 1.80 (0.41) |
|  | Treatment difference |  | -0.36 |
|  | 95% CI |  | -1.54 / 0.82 |
|  | p-value |  | 0.539 |
| *ANCOVA model* | Adjusted mean (SE) | 2.19 (0.42) | 1.77 (0.42) |
|  | Treatment difference |  | -0.43 |
|  | 95% CI |  | -1.63 / 0.78 |
|  | p-value |  | 0.479 |

**Skindex-16 Emotional**

| **Endpoint** | **Statistic** | **SOC (N=20)** | **Xonrid®+SOC (N=20)** |
| --- | --- | --- | --- |
| *Visit 7* | N | 13 | 17 |
|  | Mean (SD) | 1.14 (1.91) | 0.45 (0.64) |
|  | Median | 0.00 | 0.14 |
|  | Min - Max | 0.00 / 6.00 | 0.00 / 1.86 |
| *T-test* | Adjusted mean (SE) | 1.14 (0.37) | 0.45 (0.33) |
|  | Treatment difference |  | -0.69 |
|  | 95% CI |  | -1.70 / 0.32 |
|  | p-value |  | 0.175 |
| *ANCOVA model* | Adjusted mean (SE) | 1.15 (0.38) | 0.45 (0.33) |
|  | Treatment difference |  | -0.70 |
|  | 95% CI |  | -1.73 / 0.33 |
|  | p-value |  | 0.175 |
| *Visit 8* | N | 13 | 17 |
|  | Mean (SD) | 0.95 (1.56) | 0.43 (0.55) |
|  | Median | 0.43 | 0.14 |
|  | Min - Max | 0.00 / 5.71 | 0.00 / 1.86 |
| *T-test* | Adjusted mean (SE) | 0.95 (0.31) | 0.43 (0.27) |
|  | Treatment difference |  | -0.52 |
|  | 95% CI |  | -1.35 / 0.31 |
|  | p-value |  | 0.212 |
| *ANCOVA model* | Adjusted mean (SE) | 0.95 (0.31) | 0.43 (0.27) |
|  | Treatment difference |  | -0.52 |
|  | 95% CI |  | -1.36 / 0.33 |
|  | p-value |  | 0.221 |
| Visit 9 | N | 17 | 20 |
|  | Mean (SD) | 0.79 (1.51) | 0.24 (0.40) |
|  | Median | 0.00 | 0.07 |
|  | Min - Max | 0.00 / 4.86 | 0.00 / 1.71 |
| T-test | Adjusted mean (SE) | 0.79 (0.26) | 0.24 (0.24) |
|  | Treatment difference |  | -0.55 |
|  | 95% CI |  | -1.27 / 0.16 |
|  | p-value |  | 0.123 |
| ANCOVA model | Adjusted mean (SE) | 0.79 (0.26) | 0.24 (0.24) |
|  | Treatment difference |  | -0.55 |
|  | 95% CI |  | -1.27 / 0.17 |
|  | p-value |  | 0.127 |
|  |  |  |  |
| Worst Skindex-16 score | N | 20 | 20 |
|  | Mean (SD) | 2.16 (2.18) | 1.80 (1.45) |
|  | Median | 0.88 | 1.13 |
|  | Min - Max | 0.00 / 6.00 | 0.00 / 4.75 |
| T-test | Adjusted mean (SE) | 2.16 (0.41) | 1.80 (0.41) |
|  | Treatment difference |  | -0.36 |
|  | 95% CI |  | -1.54 / 0.82 |
|  | p-value |  | 0.539 |
|  |  |  |  |
| ANCOVA model | Adjusted mean (SE) | 2.17 (0.42) | 1.80 (0.42) |
|  | Treatment difference |  | -0.37 |
|  | 95% CI |  | -1.57 / 0.83 |
|  | p-value |  | 0.535 |

**Skindex-16 Functional**

| **Endpoint** | **Statistic** | **SOC (N=20)** | **Xonrid®+SOC (N=20)** |
| --- | --- | --- | --- |
| *Week 5* | N | 13 | 17 |
|  | Mean (SD) | 1.15 (2.10) | 0.21 (0.53) |
|  | Median | 0.00 | 0.00 |
|  | Min - Max | 0.00 / 5.40 | 0.00 / 2.00 |
| *T-test* | Adjusted mean (SE) | 1.15 (0.40) | 0.21 (0.35) |
|  | Treatment difference |  | -0.94 |
|  | 95% CI |  | -2.02 / 0.14 |
|  | p-value |  | 0.085 |
|  |  |  |  |
| *ANCOVA model* | Adjusted mean (SE) | 1.08 (0.38) | 0.27 (0.33) |
|  | Treatment difference |  | -0.82 |
|  | 95% CI |  | -1.86 / 0.22 |
|  | p-value |  | 0.119 |
|  |  |  |  |
| *Week 6* | N | 13 | 17 |
|  | Mean (SD) | 0.75 (1.39) | 0.11 (0.30) |
|  | Median | 0.00 | 0.00 |
|  | Min - Max | 0.00 / 4.80 | 0.00 / 1.00 |
| *T-test* | Adjusted mean (SE) | 0.75 (0.26) | 0.11 (0.23) |
|  | Treatment difference |  | -0.65 |
|  | 95% CI |  | -1.36 / 0.06 |
|  | p-value |  | 0.071 |
|  |  |  |  |
| *ANCOVA model* | Adjusted mean (SE) | 0.71 (0.25) | 0.14 (0.22) |
|  | Treatment difference |  | -0.57 |
|  | 95% CI |  | -1.26 / 0.11 |
|  | p-value |  | 0.099 |
|  |  |  |  |
| *2 weeks after RT end* | N | 17 | 20 |
|  | Mean (SD) | 0.62 (1.31) | 0.08 (0.31) |
|  | Median | 0.00 | 0.00 |
|  | Min - Max | 0.00 / 4.20 | 0.00 / 1.40 |
| *T-test* | Adjusted mean (SE) | 0.62 (0.22) | 0.08 (0.20) |
|  | Treatment difference |  | -0.54 |
|  | 95% CI |  | -1.16 / 0.07 |
|  | p-value |  | 0.080 |
|  |  |  |  |
| *ANCOVA model* | Adjusted mean (SE) | 0.63 (0.23) | 0.07 (0.21) |
|  | Treatment difference |  | -0.56 |
|  | 95% CI |  | -1.19 / 0.06 |
|  | p-value |  | 0.077 |
|  |  |  |  |
| *Worst Skindex-16 score* | N | 20 | 20 |
|  | Mean (SD) | 2.16 (2.18) | 1.80 (1.45) |
|  | Median | 0.88 | 1.13 |
|  | Min - Max | 0.00 / 6.00 | 0.00 / 4.75 |
| *T-test* | Adjusted mean (SE) | 2.16 (0.41) | 1.80 (0.41) |
|  | Treatment difference |  | -0.36 |
|  | 95% CI |  | -1.54 / 0.82 |
|  | p-value |  | 0.539 |
| *ANCOVA model* | Adjusted mean (SE) | 2.14 (0.42) | 1.82 (0.42) |
|  | Treatment difference |  | -0.32 |
|  | 95% CI |  | -1.51 / 0.88 |
|  | p-value |  | 0.594 |
