# Supplementary material for: A monocentric, open-label randomized standard-of-care controlled study of XONRID®, a medical device for the prevention and treatment of radiation-induced dermatitis in breast and head and neck cancer patients
Source: Radiat Oncol. 2020 Aug 13;15:193. doi: 10.1186/s13014-020-01633-0 (PMC7427075; doi:10.1186/s13014-020-01633-0)
Supplement: Supplementary file 2 — Additional file 2. Skindex-16 in HNC patients. [file 13014_2020_1633_MOESM2_ESM.docx]

**Additional file 2**

**Skindex-16 in HNC patients**

| **Endpoint** | **Statistic** | **SOC (N=20)** | **Xonrid®+SOC (N=20)** |
| --- | --- | --- | --- |
| *Visit 7* | N | 15 | 16 |
|  | Mean (SD) | 0.82 (1.07) | 1.13 (1.18) |
|  | Median | 0.50 | 0.75 |
|  | Min - Max | 0.00 / 4.00 | 0.00 / 3.00 |
| *T-test* | Adjusted mean (SE) | 0.82 (0.29) | 1.13 (0.28) |
|  | Treatment difference |  | 0.31 |
|  | 95% CI |  | -0.52 / 1.14 |
|  | p-value |  | 0.453 |
| *ANCOVA model* | Adjusted mean (SE) | 0.82 (0.29) | 1.13 (0.28) |
|  | Treatment difference |  | 0.31 |
|  | 95% CI |  | -0.52 / 1.14 |
|  | p-value |  | 0.453 |
| *Visit 8* | N | 14 | 11 |
|  | Mean (SD) | 1.48 (1.46) | 1.16 (1.42) |
|  | Median | 1.25 | 0.50 |
|  | Min - Max | 0.00 / 5.50 | 0.00 / 4.25 |
| *T-test* | Adjusted mean (SE) | 1.48 (0.39) | 1.16 (0.44) |
|  | Treatment difference |  | -0.32 |
|  | 95% CI |  | -1.53 / 0.88 |
|  | p-value |  | 0.584 |
| *ANCOVA model* | Adjusted mean (SE) | 1.48 (0.39) | 1.16 (0.44) |
|  | Treatment difference |  | -0.32 |
|  | 95% CI |  | -1.53 / 0.88 |
|  | p-value |  | 0.584 |
| *Visit 9* | N | 8 | 13 |
|  | Mean (SD) | 1.72 (1.59) | 1.19 (1.43) |
|  | Median | 1.50 | 0.75 |
|  | Min - Max | 0.00 / 5.25 | 0.00 / 4.00 |
| *T-test* | Adjusted mean (SE) | 1.72 (0.53) | 1.19 (0.41) |
|  | Treatment difference |  | -0.53 |
|  | 95% CI |  | -1.93 / 0.87 |
|  | p-value |  | 0.441 |
|  |  |  |  |
| *ANCOVA model* | Adjusted mean (SE) | 1.72 (0.53) | 1.19 (0.41) |
|  | Treatment difference |  | -0.53 |
|  | 95% CI |  | -1.93 / 0.87 |
|  | p-value |  | 0.441 |
| *Visit 10* | N | 11 | 16 |
|  | Mean (SD) | 0.93 (1.01) | 1.03 (1.58) |
|  | Median | 1.00 | 0.00 |
|  | Min - Max | 0.00 / 2.75 | 0.00 / 5.25 |
| *T-test* | Adjusted mean (SE) | 0.93 (0.42) | 1.03 (0.35) |
|  | Treatment difference |  | 0.10 |
|  | 95% CI |  | -1.02 / 1.21 |
|  | p-value |  | 0.856 |
|  |  |  |  |
| *ANCOVA model* | Adjusted mean (SE) | 0.90 (0.43) | 1.05 (0.36) |
|  | Treatment difference |  | 0.16 |
|  | 95% CI |  | -1.01 / 1.33 |
|  | p-value |  | 0.785 |
|  |  |  |  |
| *Worst Skindex-16 score* | N | 19 | 19 |
|  | Mean (SD) | 1.84 (1.63) | 1.87 (1.52) |
|  | Median | 1.75 | 1.50 |
|  | Min - Max | 0.00 / 5.75 | 0.00 / 5.25 |
| *T-test* | Adjusted mean (SE) | 1.84 (0.36) | 1.87 (0.36) |
|  | Treatment difference |  | 0.03 |
|  | 95% CI |  | -1.01 / 1.06 |
|  | p-value |  | 0.959 |
| *ANCOVA model* | Adjusted mean (SE) | 1.85 (0.37) | 1.86 (0.37) |
|  | Treatment difference |  | 0.01 |
|  | 95% CI |  | -1.06 / 1.07 |
|  | p-value |  | 0.989 |

**Skindex-16 Emotional**

| **Endpoint** | **Statistic** | **SOC (N=20)** | **Xonrid®+SOC (N=20)** |
| --- | --- | --- | --- |
| *Visit 7* | N | 15 | 16 |
|  | Mean (SD) | 0.36 (0.71) | 0.58 (1.01) |
|  | Median | 0.14 | 0.14 |
|  | Min - Max | 0.00 / 2.71 | 0.00 / 3.57 |
| *T-test* | Adjusted mean (SE) | 0.36 (0.23) | 0.58 (0.22) |
|  | Treatment difference |  | 0.22 |
|  | 95% CI |  | -0.43 / 0.86 |
|  | p-value |  | 0.494 |
| *ANCOVA model* | Adjusted mean (SE) | 0.40 (0.24) | 0.55 (0.23) |
|  | Treatment difference |  | 0.15 |
|  | 95% CI |  | -0.54 / 0.84 |
|  | p-value |  | 0.653 |
| *Visit 8* | N | 14 | 11 |
|  | Mean (SD) | 0.94 (1.23) | 0.47 (1.00) |
|  | Median | 0.57 | 0.00 |
|  | Min - Max | 0.00 / 4.43 | 0.00 / 3.00 |
| *T-test* | Adjusted mean (SE) | 0.94 (0.30) | 0.47 (0.34) |
|  | Treatment difference |  | -0.47 |
|  | 95% CI |  | -1.42 / 0.48 |
|  | p-value |  | 0.316 |
| *ANCOVA model* | Adjusted mean (SE) | 1.00 (0.32) | 0.39 (0.36) |
|  | Treatment difference |  | -0.61 |
|  | 95% CI |  | -1.64 / 0.42 |
|  | p-value |  | 0.231 |
| *Visit 9* | N | 8 | 13 |
|  | Mean (SD) | 1.29 (2.00) | 0.71 (1.28) |
|  | Median | 0.29 | 0.14 |
|  | Min - Max | 0.00 / 5.57 | 0.00 / 4.57 |
| *T-test* | Adjusted mean (SE) | 1.29 (0.56) | 0.71 (0.44) |
|  | Treatment difference |  | -0.57 |
|  | 95% CI |  | -2.06 / 0.92 |
|  | p-value |  | 0.432 |
| *ANCOVA model* | Adjusted mean (SE) | 1.38 (0.58) | 0.65 (0.45) |
|  | Treatment difference |  | -0.73 |
|  | 95% CI |  | -2.29 / 0.83 |
|  | p-value |  | 0.338 |
| *Visit 10* | N | 11 | 16 |
|  | Mean (SD) | 1.08 (1.63) | 0.58 (0.95) |
|  | Median | 0.14 | 0.00 |
|  | Min - Max | 0.00 / 5.14 | 0.00 / 2.57 |
| *T-test* | Adjusted mean (SE) | 1.08 (0.38) | 0.58 (0.32) |
|  | Treatment difference |  | -0.50 |
|  | 95% CI |  | -1.52 / 0.52 |
|  | p-value |  | 0.324 |
| *ANCOVA model* | Adjusted mean (SE) | 0.74 (0.27) | 0.81 (0.23) |
|  | Treatment difference |  | 0.07 |
|  | 95% CI |  | -0.68 / 0.82 |
|  | p-value |  | 0.856 |
| *Worst Skindex-16 score* | N | 19 | 19 |
|  | Mean (SD) | 1.84 (1.63) | 1.87 (1.52) |
|  | Median | 1.75 | 1.50 |
|  | Min - Max | 0.00 / 5.75 | 0.00 / 5.25 |
| *T-test* | Adjusted mean (SE) | 1.84 (0.36) | 1.87 (0.36) |
|  | Treatment difference |  | 0.03 |
|  | 95% CI |  | -1.01 / 1.06 |
|  | p-value |  | 0.959 |
| *ANCOVA model* | Adjusted mean (SE) | 2.04 (0.36) | 1.67 (0.36) |
|  | Treatment difference |  | -0.37 |
|  | 95% CI |  | -1.42 / 0.69 |
|  | p-value |  | 0.488 |

**Skindex-16 Functional**

| **Endpoint** | **Statistic** | **SOC (N=20)** | **Xonrid®+SOC (N=20)** |
| --- | --- | --- | --- |
| *Visit 7* | N | 15 | 16 |
|  | Mean (SD) | 0.12 (0.32) | 0.29 (0.66) |
|  | Median | 0.00 | 0.00 |
|  | Min - Max | 0.00 / 1.00 | 0.00 / 2.20 |
| *T-test* | Adjusted mean (SE) | 0.12 (0.13) | 0.29 (0.13) |
|  | Treatment difference |  | 0.17 |
|  | 95% CI |  | -0.22 / 0.55 |
|  | p-value |  | 0.379 |
|  |  |  |  |
| *ANCOVA model* | Adjusted mean (SE) | 0.13 (0.14) | 0.28 (0.13) |
|  | Treatment difference |  | 0.15 |
|  | 95% CI |  | -0.25 / 0.54 |
|  | p-value |  | 0.450 |
| *Visit 8* | N | 14 | 11 |
|  | Mean (SD) | 0.46 (0.71) | 0.42 (1.01) |
|  | Median | 0.10 | 0.00 |
|  | Min - Max | 0.00 / 2.40 | 0.00 / 3.20 |
| *T-test* | Adjusted mean (SE) | 0.46 (0.23) | 0.42 (0.26) |
|  | Treatment difference |  | -0.04 |
|  | 95% CI |  | -0.75 / 0.67 |
|  | p-value |  | 0.911 |
| *ANCOVA model* | Adjusted mean (SE) | 0.52 (0.24) | 0.34 (0.27) |
|  | Treatment difference |  | -0.18 |
|  | 95% CI |  | -0.95 / 0.58 |
|  | p-value |  | 0.625 |
| *Visit 9* | N | 8 | 13 |
|  | Mean (SD) | 1.00 (1.96) | 0.48 (1.32) |
|  | Median | 0.00 | 0.00 |
|  | Min - Max | 0.00 / 5.40 | 0.00 / 4.60 |
| *T-test* | Adjusted mean (SE) | 1.00 (0.56) | 0.48 (0.44) |
|  | Treatment difference |  | -0.52 |
|  | 95% CI |  | -2.01 / 0.97 |
|  | p-value |  | 0.472 |
| *ANCOVA model* | Adjusted mean (SE) | 1.00 (0.57) | 0.48 (0.45) |
|  | Treatment difference |  | -0.52 |
|  | 95% CI |  | -2.05 / 1.00 |
|  | p-value |  | 0.480 |
| *Visit 10* | N | 11 | 16 |
|  | Mean (SD) | 0.80 (1.80) | 0.43 (0.81) |
|  | Median | 0.00 | 0.00 |
|  | Min - Max | 0.00 / 6.00 | 0.00 / 2.20 |
| *T-test* | Adjusted mean (SE) | 0.80 (0.39) | 0.43 (0.32) |
|  | Treatment difference |  | -0.38 |
|  | 95% CI |  | -1.42 / 0.67 |
|  | p-value |  | 0.468 |
| *ANCOVA model* | Adjusted mean (SE) | 0.57 (0.25) | 0.58 (0.20) |
|  | Treatment difference |  | 0.01 |
|  | 95% CI |  | -0.65 / 0.68 |
|  | p-value |  | 0.970 |
| *Worst Skindex-16 score* | N | 19 | 19 |
|  | Mean (SD) | 1.84 (1.63) | 1.87 (1.52) |
|  | Median | 1.75 | 1.50 |
|  | Min - Max | 0.00 / 5.75 | 0.00 / 5.25 |
| *T-test* | Adjusted mean (SE) | 1.84 (0.36) | 1.87 (0.36) |
|  | Treatment difference |  | 0.03 |
|  | 95% CI |  | -1.01 / 1.06 |
|  | p-value |  | 0.959 |
| *ANCOVA model* | Adjusted mean (SE) | 1.95 (0.36) | 1.76 (0.36) |
|  | Treatment difference |  | -0.19 |
|  | 95% CI |  | -1.23 / 0.85 |
|  | p-value |  | 0.710 |
