# Supplementary material for: A monocentric, open-label randomized standard-of-care controlled study of XONRID®, a medical device for the prevention and treatment of radiation-induced dermatitis in breast and head and neck cancer patients
Source: Radiat Oncol. 2020 Aug 13;15:193. doi: 10.1186/s13014-020-01633-0 (PMC7427075; doi:10.1186/s13014-020-01633-0)
Supplement: Supplementary file 3 — Additional file 3. AEs in BC patients. [file 13014_2020_1633_MOESM3_ESM.docx]

**Additional file 3- AEs in BC patients**

| **AE details** | **Statistic** | **SOC (N=20)** | **Xonrid®++SOC (N=20)** |
| --- | --- | --- | --- |
| *Have any AE occurred?* | NO | 7 ( 35.0%) | 4 ( 20.0%) |
|  | YES | 13 ( 65.0%) | 16 ( 80.0%) |
|  | Odds Ratio |  |  |
|  | 95% CI |  |  |
|  | p-value |  |  |
| *Number of AE per subject* | N | 13 | 16 |
|  | Mean (SD) | 1.77 (0.93) | 1.81 (1.05) |
|  | Median | 2.00 | 1.00 |
|  | Min - Max | 1.00 / 4.00 | 1.00 / 4.00 |
|  | Adjusted mean (SE) | 1.77 (0.28) | 1.81 (0.25) |
|  | Treatment difference | . | 0.04 |
|  | 95% CI |  | -0.72 / 0.81 |
|  | p-value |  | 0.9082 |
| *Total number of adverse events^* | N | (N=23) | (N=29) |
| *Relatedness with study treatment* | NONE | 23 (100.0%) | 28 ( 96.6%) |
|  | POSSIBLE |  | 1 ( 3.4%) |
| *Severity* | MILD | 20 ( 87.0%) | 25 ( 86.2%) |
|  | MODERATE | 3 ( 13.0%) | 3 ( 10.3%) |
|  | SEVERE |  | 1 ( 3.4%) |
|  | p-value |  | 0.6457 |
| *Seriousness* | NO | 23 (100.0%) | 29 (100.0%) |
|  | p-value |  | n.a. |

| **System Organ Class (SOC) . Preferred Term (PT)** | **SOC (N=20)** | | | | **Xonrid®++SOC (N=20)** | | |
| --- | --- | --- | --- | --- | --- | --- | --- |
|  | ***Event*** | | ***Patients*** | ***(%)*** | ***Event*** | ***Patients*** | ***(%)*** |
| **OVERALL** | 23 | | 13 | 65.00 | 29 | 16 | 80.00 |
| Ear and labyrinth disorders  *. Ear infection* | 1 | | 1 | 5.00 | 0 | 0 | 0.00 |
|  | 1 | | 1 | 5.00 | 0 | 0 | 0.00 |
| General disorders and administration site conditions  *. Asthenia*  *. Axillary pain*  *. Burning sensation*  *. Effusion*  *. Gastrointestinal motility disorder*  *. Hot flush*  *. Oedema*  *. Pyrexia* | 8 | | 8 | 40.00 | 8 | 7 | 35.00 |
|  | 2 | | 2 | 10.00 | 1 | 1 | 5.00 |
|  | 0 | | 0 | 0.00 | 1 | 1 | 5.00 |
|  | 1 | | 1 | 5.00 | 0 | 0 | 0.00 |
|  | 0 | | 0 | 0.00 | 1 | 1 | 5.00 |
|  | 0 | | 0 | 0.00 | 1 | 1 | 5.00 |
|  | 1 | | 1 | 5.00 | 0 | 0 | 0.00 |
|  | 4 | | 4 | 20.00 | 2 | 2 | 10.00 |
|  | 0 | | 0 | 0.00 | 2 | 2 | 10.00 |
| Infections and infestations  *. Influenza* | 1 | | 1 | 5.00 | 0 | 0 | 0.00 |
|  | 1 | | 1 | 5.00 | 0 | 0 | 0.00 |
| Injury, poisoning and procedural complications  *. Wound secretion* | 0 | | 0 | 0.00 | 1 | 1 | 5.00 |
|  | 0 | | 0 | 0.00 | 1 | 1 | 5.00 |
| Musculoskeletal and connective tissue disorders  *. Arthralgia*  *. Musculoskeletal pain*  *. Myalgia*  *. Neck pain*  *. Pain in extremity* | 1 | | 1 | 5.00 | 5 | 4 | 20.00 |
|  | 0 | | 0 | 0.00 | 1 | 1 | 5.00 |
|  | 0 | | 0 | 0.00 | 1 | 1 | 5.00 |
|  | 0 | | 0 | 0.00 | 1 | 1 | 5.00 |
|  | 1 | | 1 | 5.00 | 1 | 1 | 5.00 |
|  | 0 | | 0 | 0.00 | 1 | 1 | 5.00 |
| Nervous system disorders  *. Headache*  *. Hyperaesthesia* | 0 | | 0 | 0.00 | 2 | 2 | 10.00 |
|  | 0 | | 0 | 0.00 | 1 | 1 | 5.00 |
|  | 0 | | 0 | 0.00 | 1 | 1 | 5.00 |
| Renal and urinary disorders  *. Pelvic pain* | 1 | | 1 | 5.00 | 0 | 0 | 0.00 |
|  | 1 | | 1 | 5.00 | 0 | 0 | 0.00 |
| Reproductive system and breast disorders  *. Breast mass*  *. Breast pain* | 3 | | 3 | 15.00 | 0 | 0 | 0.00 |
|  | 1 | | 1 | 5.00 | 0 | 0 | 0.00 |
|  | 2 | | 2 | 10.00 | 0 | 0 | 0.00 |
| Skin and subcutaneous tissue disorders  *. Dermatitis*  *. Erythema*  *. Pruritus*  *. Scab*  *. Scar* | 8 | | 7 | 35.00 | 13 | 11 | 55.00 |
|  | 1 | | 1 | 5.00 | 0 | 0 | 0.00 |
|  | 0 | | 0 | 0.00 | 1 | 1 | 5.00 |
|  | 5 | | 4 | 20.00 | 6 | 6 | 30.00 |
|  | 0 | 0 | | 0.00 | 1 | 1 | 5.00 |
|  | 2 | 2 | | 10.00 | 5 | 4 | 20.00 |
