# Supplementary material for: A monocentric, open-label randomized standard-of-care controlled study of XONRID®, a medical device for the prevention and treatment of radiation-induced dermatitis in breast and head and neck cancer patients
Source: Radiat Oncol. 2020 Aug 13;15:193. doi: 10.1186/s13014-020-01633-0 (PMC7427075; doi:10.1186/s13014-020-01633-0)
Supplement: Supplementary file 4 — Additional file 4. AEs in HNC patients. [file 13014_2020_1633_MOESM4_ESM.docx]

**Additional file 4 - AEs in HNC patients**

| **AE details** | **Statistic** | **SOC (N=20)** | **XONRID + SOC (N=20)** |
| --- | --- | --- | --- |
| *Have any AE occurred?* | NO | 3 ( 15.0%) |  |
|  | YES | 17 ( 85.0%) | 20 (100.0%) |
|  | Odds Ratio |  |  |
|  | 95% CI |  |  |
|  | p-value |  |  |
| *Number of AE per subject* | N | 17 | 20 |
|  | Mean (SD) | 12.94 (7.20) | 14.10 (8.53) |
|  | Median | 11.00 | 14.50 |
|  | Min - Max | 1.00 / 30.00 | 4.00 / 29.00 |
|  | Adjusted mean (SE) | 12.94 (1.93) | 14.10 (1.78) |
|  | Treatment difference |  | 1.16 |
|  | 95% CI |  | -4.16 / 6.48 |
|  | p-value |  | 0.6612 |
| *Total number of adverse events^* | N | (N=220) | (N=282) |
| *Relatedness with study treatment* | NONE | 220 (100.0%) | 282 (100.0%) |
| *Severity* | MILD | 128 ( 58.2%) | 166 ( 58.9%) |
|  | MODERATE | 81 ( 36.8%) | 103 ( 36.5%) |
|  | SEVERE | 11 ( 5.0%) | 13 ( 4.6%) |
|  | p-value |  | 0.9743 |
| *Seriousness* | NO | 220 (100.0%) | 282 (100.0%) |
|  | p-value |  | n.a. |

| **System Organ Class (SOC) . Preferred Term (PT)** | **SOC (N=20)** | | | **Xonrid®+SOC (N=20)** | | |
| --- | --- | --- | --- | --- | --- | --- |
|  | **Event** | **Patients** | **(%)** | **Event** | **Patients** | **(%)** |
| **OVERALL** | 220 | 17 | 85.00 | 282 | 20 | 100.00 |
| Blood and lymphatic system disorders | 1 | 1 | 5.00 | 1 | 1 | 5.00 |
| *. Lymphoedema* | 0 | 0 | 0.00 | 1 | 1 | 5.00 |
| *. Thrombocytopenia* | 1 | 1 | 5.00 | 0 | 0 | 0.00 |
| Ear and labyrinth disorders | 2 | 2 | 10.00 | 7 | 5 | 25.00 |
| *. Deafness* | 0 | 0 | 0.00 | 1 | 1 | 5.00 |
| *. Ear pain* | 1 | 1 | 5.00 | 0 | 0 | 0.00 |
| *. Hypoacusis* | 0 | 0 | 0.00 | 2 | 2 | 10.00 |
| *. Tinnitus* | 1 | 1 | 5.00 | 3 | 3 | 15.00 |
| *. Vertigo* | 0 | 0 | 0.00 | 1 | 1 | 5.00 |
| Eye disorders | 3 | 3 | 15.00 | 2 | 2 | 10.00 |
| . Conjunctivitis | 2 | 2 | 10.00 | 0 | 0 | 0.00 |
| . Eye irritation | 0 | 0 | 0.00 | 1 | 1 | 5.00 |
| . Lacrimation disorders | 0 | 0 | 0.00 | 1 | 1 | 5.00 |
| . Xerophthalmia | 1 | 1 | 5.00 | 0 | 0 | 0.00 |
| Gastrointestinal disorders | 157 | 17 | 85.00 | 194 | 20 | 100.00 |
| *. Ageusia* | 3 | 3 | 15.00 | 2 | 2 | 10.00 |
| *. Aphthous ulcer* | 1 | 1 | 5.00 | 3 | 3 | 15.00 |
| *. Constipation* | 1 | 1 | 5.00 | 4 | 4 | 20.00 |
| *. Dysgeusia* | 20 | 12 | 60.00 | 25 | 18 | 90.00 |
| *. Dysphagia* | 32 | 15 | 75.00 | 24 | 14 | 70.00 |
| *. Glossodynia* | 1 | 1 | 5.00 | 2 | 2 | 10.00 |
| *. Hiccups* | 0 | 0 | 0.00 | 1 | 1 | 5.00 |
| *. Nausea* | 7 | 7 | 35.00 | 17 | 9 | 45.00 |
| *. Odynophagia* | 19 | 10 | 50.00 | 19 | 13 | 65.00 |
| *. Oesophageal candidiasis* | 1 | 1 | 5.00 | 0 | 0 | 0.00 |
| *. Oral pain* | 1 | 1 | 5.00 | 5 | 3 | 15.00 |
| *. Paraesthesia oral* | 0 | 0 | 0.00 | 1 | 1 | 5.00 |
| *. Salivary hypersecretion* | 0 | 0 | 0.00 | 3 | 3 | 15.00 |
| *. Sore throat G1* | 0 | 0 | 0.00 | 1 | 1 | 5.00 |
| *. Sore throat G10* | 1 | 1 | 5.00 | 0 | 0 | 0.00 |
| *. Sore throat G11* | 0 | 0 | 0.00 | 1 | 1 | 5.00 |
| *. Sore throat G12* | 1 | 1 | 5.00 | 0 | 0 | 0.00 |
| *. Sore throat G13* | 0 | 0 | 0.00 | 1 | 1 | 5.00 |
| *. Sore throat G2* | 1 | 1 | 5.00 | 0 | 0 | 0.00 |
| *. Sore throat G3* | 0 | 0 | 0.00 | 1 | 1 | 5.00 |
| *. Sore throat G4* | 1 | 1 | 5.00 | 0 | 0 | 0.00 |
| *. Sore throat G5* | 0 | 0 | 0.00 | 1 | 1 | 5.00 |
| *. Sore throat G6* | 0 | 0 | 0.00 | 1 | 1 | 5.00 |
| *. Sore throat G7* | 0 | 0 | 0.00 | 1 | 1 | 5.00 |
| *. Sore throat G8* | 1 | 1 | 5.00 | 0 | 0 | 0.00 |
| *. Sore throat G9* | 1 | 1 | 5.00 | 0 | 0 | 0.00 |
| *. Stomatitis* | 32 | 16 | 80.00 | 46 | 19 | 95.00 |
| *. Tongue ulceration* | 1 | 1 | 5.00 | 1 | 1 | 5.00 |
| *. Tooth abscess* | 1 | 1 | 5.00 | 0 | 0 | 0.00 |
| *. Trichoglossia* | 1 | 1 | 5.00 | 0 | 0 | 0.00 |
| *. Vomiting* | 0 | 0 | 0.00 | 3 | 3 | 15.00 |
| *. Xerostomia* | 30 | 15 | 75.00 | 31 | 19 | 95.00 |
| General disorders and administration site conditions | 22 | 13 | 65.00 | 23 | 12 | 60.00 |
| *. Asthenia* | 17 | 12 | 60.00 | 18 | 9 | 45.00 |
| *. Enanthema* | 1 | 1 | 5.00 | 0 | 0 | 0.00 |
| *. Mucosal dryness* | 1 | 1 | 5.00 | 0 | 0 | 0.00 |
| *. Mucosal inflammation* | 1 | 1 | 5.00 | 2 | 2 | 10.00 |
| *. Mucosal pain* | 0 | 0 | 0.00 | 1 | 1 | 5.00 |
| *. Pain* | 1 | 1 | 5.00 | 2 | 2 | 10.00 |
| *. Pyrexia* | 1 | 1 | 5.00 | 0 | 0 | 0.00 |
| Infections and infestations | 7 | 7 | 35.00 | 13 | 12 | 60.00 |
| *. Candida infection* | 4 | 4 | 20.00 | 11 | 10 | 50.00 |
| *. Oral candidiasis* | 1 | 1 | 5.00 | 2 | 2 | 10.00 |
| *. Oropharyngeal candidiasis* | 2 | 2 | 10.00 | 0 | 0 | 0.00 |
| Investigations | 1 | 1 | 5.00 | 10 | 7 | 35.00 |
| *. C-reactive protein increased* | 0 | 0 | 0.00 | 5 | 5 | 25.00 |
| *. Haemoglobin decreased* | 0 | 0 | 0.00 | 2 | 2 | 10.00 |
| *. Platelet count decreased* | 0 | 0 | 0.00 | 1 | 1 | 5.00 |
| *. Red blood cell count decreased* | 0 | 0 | 0.00 | 1 | 1 | 5.00 |
| *. Weight decreased* | 1 | 1 | 5.00 | 0 | 0 | 0.00 |
| *. White blood cell count decreased* | 0 | 0 | 0.00 | 1 | 1 | 5.00 |
| Metabolism and nutrition disorders | 1 | 1 | 5.00 | 1 | 1 | 5.00 |
| *. Hyperkalemia* | 1 | 1 | 5.00 | 0 | 0 | 0.00 |
| *. Iron deficiency* | 0 | 0 | 0.00 | 1 | 1 | 5.00 |
| Musculoskeletal and connective tissue disorders | 2 | 2 | 10.00 | 2 | 1 | 5.00 |
| *. Back pain* | 2 | 2 | 10.00 | 0 | 0 | 0.00 |
| *. Neck pain* | 0 | 0 | 0.00 | 1 | 1 | 5.00 |
| *. Pain in extremity* | 0 | 0 | 0.00 | 1 | 1 | 5.00 |
| Nervous system disorders | 0 | 0 | 0.00 | 3 | 3 | 15.00 |
| *. Dysarthria* | 0 | 0 | 0.00 | 2 | 2 | 10.00 |
| *. Paraesthesia* | 0 | 0 | 0.00 | 1 | 1 | 5.00 |
| Renal and urinary disorders | 0 | 0 | 0.00 | 1 | 1 | 5.00 |
| *. Acute kidney injury* | 0 | 0 | 0.00 | 1 | 1 | 5.00 |
| Reproductive system and breast disorders | 2 | 2 | 10.00 | 0 | 0 | 0.00 |
| *. Benign prostatic hyperplasia* | 1 | 1 | 5.00 | 0 | 0 | 0.00 |
| *. Cervix oedema* | 1 | 1 | 5.00 | 0 | 0 | 0.00 |
| Respiratory, thoracic and mediastinal disorders | 12 | 7 | 35.00 | 15 | 8 | 40.00 |
| *. Cough* | 3 | 3 | 15.00 | 4 | 3 | 15.00 |
| *. Dysphonia* | 6 | 5 | 25.00 | 4 | 3 | 15.00 |
| *. Increased upper airway secretion* | 1 | 1 | 5.00 | 3 | 3 | 15.00 |
| *. Nasal crusting* | 1 | 1 | 5.00 | 0 | 0 | 0.00 |
| *. Parosmia* | 0 | 0 | 0.00 | 1 | 1 | 5.00 |
| *. Pharyngeal haemorrhage* | 0 | 0 | 0.00 | 1 | 1 | 5.00 |
| *. Pharyngeal inflammation* | 0 | 0 | 0.00 | 1 | 1 | 5.00 |
| *. Rhinorrhoea* | 1 | 1 | 5.00 | 0 | 0 | 0.00 |
| *. Tracheal inflammation* | 0 | 0 | 0.00 | 1 | 1 | 5.00 |
| Skin and subcutaneous tissue disorders | 10 | 8 | 40.00 | 6 | 5 | 25.00 |
| *. Alopecia* | 1 | 1 | 5.00 | 2 | 2 | 10.00 |
| *. Dermatitis acneiform* | 1 | 1 | 5.00 | 0 | 0 | 0.00 |
| *. Erythema* | 1 | 1 | 5.00 | 0 | 0 | 0.00 |
| *. Hyperhidrosis* | 1 | 1 | 5.00 | 0 | 0 | 0.00 |
| *. Pruritus* | 6 | 6 | 30.00 | 2 | 2 | 10.00 |
| *. Scar* | 0 | 0 | 0.00 | 2 | 2 | 10.00 |
| Vascular disorders | 0 | 0 | 0.00 | 4 | 4 | 20.00 |
| *. Epistaxis* | 0 | 0 | 0.00 | 1 | 1 | 5.00 |
| *. Haemoptysis* | 0 | 0 | 0.00 | 1 | 1 | 5.00 |
| *. Hypertension* | 0 | 0 | 0.00 | 2 | 2 | 10.00 |
